# Leveraging Temporal Information to Improve Machine Learning-Based Calibration Techniques for Low-Cost Air Quality Sensors

**DOI:** 10.3390/s24092930

**Published:** 2024-05-04

**Authors:** Sharafat Ali, Fakhrul Alam, Johan Potgieter, Khalid Mahmood Arif

**Affiliations:** 1 Department of Mechanical and Electrical Engineering, Massey University, Auckland 0632, New Zealand; s.ali3@massey.ac.nz (S.A.); k.arif@massey.ac.nz (K.M.A.); 2 Department of Electrical & Electronic Engineering, Auckland University of Technology, Auckland 1010, New Zealand; 3 Manawatu Agrifood Digital Lab, Palmerston North 4410, New Zealand; j.potgieter@mafdigitallab.co.nz

**Keywords:** air quality monitoring, calibration, low-cost sensor, machine learning

## Abstract

Low-cost ambient sensors have been identified as a promising technology for monitoring air pollution at a high spatio-temporal resolution. However, the pollutant data captured by these cost-effective sensors are less accurate than their conventional counterparts and require careful calibration to improve their accuracy and reliability. In this paper, we propose to leverage temporal information, such as the duration of time a sensor has been deployed and the time of day the reading was taken, in order to improve the calibration of low-cost sensors. This information is readily available and has so far not been utilized in the reported literature for the calibration of cost-effective ambient gas pollutant sensors. We make use of three data sets collected by research groups around the world, who gathered the data from field-deployed low-cost CO and NO_2_ sensors co-located with accurate reference sensors. Our investigation shows that using the temporal information as a co-variate can significantly improve the accuracy of common machine learning-based calibration techniques, such as Random Forest and Long Short-Term Memory.

## 1. Introduction

Air pollution adversely affects public health and quality of life [1]. Therefore, researchers from a diverse range of disciplines are working on mitigating the impact of air pollution [2,3]. Monitoring outdoor air pollution is one of the means to ensure public health and safety, raise public awareness and build a sustainable urban environment [4]. The conventional sensors used for monitoring air pollutants are typically expensive and large [5]. As a result, deploying a large number of monitoring stations is not affordable, leading to a poor spatial resolution of urban pollution data. Low-Cost Sensor (LCS) technologies aim to address this challenge and make air quality monitoring with high spatio-temporal resolution feasible [6]. Many cities are adopting this approach to improve their pollutant measurement capacity [7,8,9,10].

The pollutant data captured by the LCSs are less accurate than their conventional (and expensive) counterparts [11,12]. Many innovative methods have been proposed to improve the accuracy and the operability of the LCSs [12,13]. It should be noted that the detection limits of the LCSs depend on the sensors’ hardware and how the sensors were assembled rather than the calibration techniques working on a sensor’s output. Therefore, while the accuracy of LCSs can be improved with calibration, the detection limits of such sensors cannot be increased.

A popular method for calibration is to co-locate an LCS with a high-quality sensor (reference sensor) and use the data from the reference sensor as the ground truth to derive a calibration model [7,8,9,10] for the LCS to improve its accuracy. Many regression-based calibration methods have been proposed to improve the accuracy and reliability of LCSs [13,14]. Multiple Linear Regression (MLR) [7,8,9,15,16,17,18,19], Support Vector Regression (SVR) [20,21,22,23,24,25], Random Forest Regression (RFR) [20,22,26,27,28,29,30], Neural Networks (NN) (like Multilayer Perceptron (MLP)) [8,9,10,23,24,25,29] and Recurrent Neural Networks (RNN) [23,24,25,31] are among the most common techniques reported in the literature. In this study, we selected Random Forest Regression (RFR) from the ensemble Machine Learning (ML) techniques and Long Short-Term Memory (LSTM) from the RNN-based technique as representative examples from the two most popular ML-based calibration techniques. We show how readily available and previously unexploited co-variate data can significantly improve calibration accuracy.

Random Forest Regression constructs a set of decision trees from the training dataset to infer predictions. Each level of the decision tree splits the training data into smaller subsets to predict the target value (reference reading for gas sensor calibration). This splitting process ends when the model performance does not improve further or a terminal node is reached [26]. RFR-based calibration techniques have performed well for LCSs measuring ambient gas pollutants. Examples of RFR improving the calibration of field-deployed LCSs measuring ambient gas pollutants can be found in the works of Borrego et al. [30] (CO, NO_2_, O_3_ and SO_2_), Cordero et al. [20] (NO_2_), Bigi et al. [22] (NO and NO_2_), Malings et al. [29] (CO, NO, NO_2_ and O_3_) and Zimmerman et al. [26] (CO, CO_2_, NO_2_, O_3_). One of the main reasons for RFR being utilized by many reported works is its ability to account for cross-sensitivity [26], the influence of gases other than the target pollutant on the LCS.

Many researchers have used Neural Networks to calibrate LCS data [24,32]. Unlike other NNs that mostly use current data, RNNs model the historical time series behavior present in the dataset. They have been used by Sheik et al. [33], Wang et al. [34] and Fonollosa et al. [31] for calibrating LCSs under laboratory conditions. Esposito et al. [24,25] studied multiple calibration techniques, including RNN, on different LCSs and compared their performances. It should be noted that RNN models face two issues during calibration: Firstly, the determination of time lag must be made in advance, which requires a considerable number of experiments to identify. Secondly, these RNNs fail to capture long-time dependencies in the training dataset. Therefore, Long Short-Term Memory network (LSTM), a variant of RNNs, was introduced [35]. LSTM has been used for calibrating low-cost ambient gas sensors by different research groups. Examples of such applications can be found in the works of Han et al. (CO, NO_2_, O_3_ and SO_2_) [35] and Peng et al. (NO_2_) [36], among others.

The response of the LCSs are highly susceptible to cross-sensitivity from other ambient gases [7,24] and temperature and relative humidity [7,8]. Therefore, temperature, relative humidity and cross pollutant data are traditionally used as the regressor co-variates to correct the sensor output and make the pollutant readings more accurate [7,8,9,37]. These data are usually available, as LCSs are often deployed as an array or a suite with multiple pollutant sensors along with temperature and humidity sensors.

It is well known that LCS performance drifts and degrades over time. We hypothesize that using the number of days an LCS has been deployed in operation can be used as a co-variate to enable the ML algorithms to model and address the gradual degradation. Many gas pollutants come from anthropogenic sources and are direct results of human activities (e.g., CO, NO_X_ resulting from automobile emissions) [23,38]. Therefore, it is reasonable to assume that the time of the day that influences relevant human activities will also impact the pollutant concentration and can potentially be used as a co-variate. However, the literature does not show any evidence of utilizing these parameters, which are readily available without any additional cost, for multi-variate calibration of LCSs. In this article, we demonstrate that including these parameters as input features can significantly improve the accuracy of the LCSs.

## 2. Dataset Description

**Figure 1:**
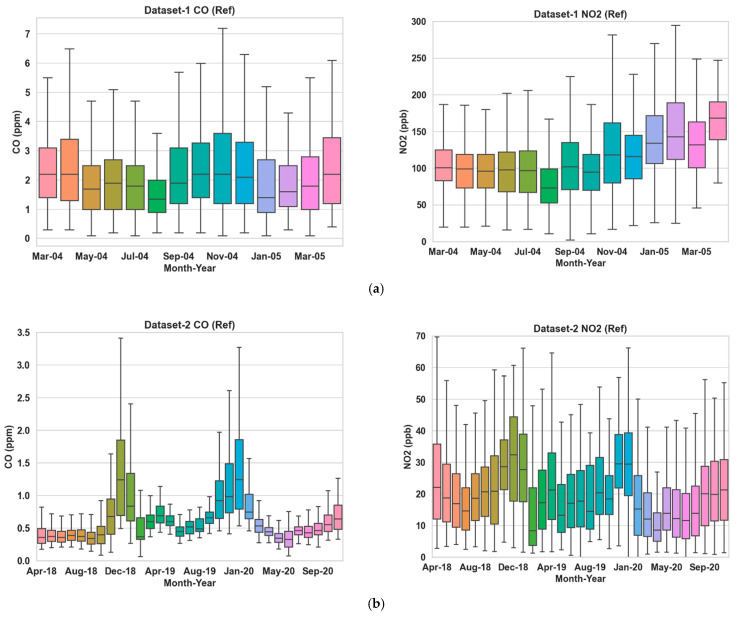

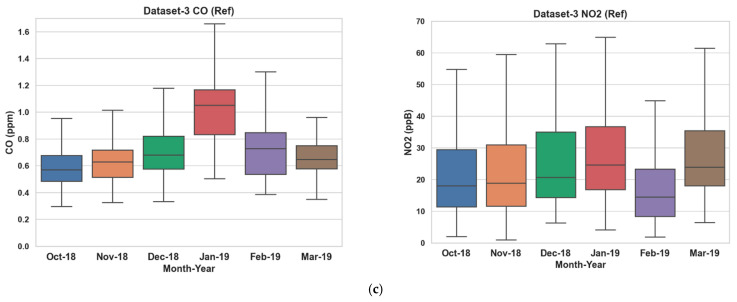
Box plots of the target pollutant concentrations as recorded by the reference sensors for Dataset 1, 2 and 3 in (**a**–**c**), respectively. The median and standard deviation of the CO readings are (1.66, 1.26), (0.49, 0.40) and (0.67, 0.25) in ppm, respectively, for the three datasets. The median and standard deviation of the NO_2_ readings for the three datasets are (109, 47.23), (18.16, 12.68) and (20.33, 15.65) in ppb, respectively.

We have focused on the calibration of an LCS measuring two gas pollutants, CO and NO_2_, for this work. Both pollutants are components of the Air Quality Index (AQI) [39]. We have utilized three datasets collected by researchers using LCSs deployed in different parts of the world. Figure 1 shows the box plot of the target pollutant (CO and NO_2_) concentrations recorded by the reference sensors for all three datasets.

The raw pollutant readings from the LCSs (unchanged electrode data) and ground truth from co-located accurate reference-grade sensors are available for all three deployments. These datasets also include other pollutant data that have allowed us to address cross-sensitivity. Temperature and relative humidity data from sensors onboard the LCSs, available for all three setups, help mitigate their respective effects. Table 1 provides a summary of the three datasets. For more details of the datasets, sensors, deployment setup, and other relevant information, please refer to the works reported in [7,10,23], as well as our previous work [40].

## 3. Methodology

### 3.1. Calibration Models

The calibration models are regressors so that,

(1) $$P_{calibrated}=\Phi \left\{P_{raw},\underline{X}\right\}.$$

Here $P_{calibrated}$ is the calibrated CO or NO_2_ reading computed from the raw readings ($P_{raw})$ of the LCS (${CO}_{raw}$ or$\;{{NO}_{2}}_{raw}$, working electrode data and/or auxiliary electrode data), as well as $\underline{X}$, which comprises co-variates. Additionally, Φ is the regression model, the parameters of which are derived from the training data in order to minimize the Mean Square Error (MSE) between the calibrated output and the ground truth received from the reference sensor. Four different scenarios have been considered for each of the ML algorithms.

#### 3.1.1. Scenario 1 (S1)

Here the co-variates are temperature, relative humidity and other pollutant readings from the LCS sensor array so that,

(2) $$P_{calibrated}^{S1}=\Phi^{\;S1}\;\left\{P_{raw},T,\;RH,{GAS}_{raw}\right\}.$$

The regressor, $\Phi^{S1}$, is derived based on *P_raw_*, the raw pollutant sensor input (working electrode data and/or auxiliary electrode data), along with temperature (*T*) and relative humidity (*RH*) readings and other pollutant readings (${GAS}_{raw}$), to minimize the MSE between $P_{calibrated}^{S1}$ and the ground truth.

#### 3.1.2. Scenario 2 (S2)

For the second scenario, $N_{day}$, the number of days the LCS has been deployed in the field is used as an additional co-variate for estimating the regressor model $\Phi^{\;S2}$. The calibrated output is

(3) $$P_{calibrated}^{S2}=\Phi^{\;S2}\;\left\{P_{raw},T,\;RH,{GAS}_{raw},N_{day}\right\}.$$

#### 3.1.3. Scenario 3 (S3)

In Scenario 3, $N_{day}$ is replaced with Hour, the time of the day the readings were taken at for estimating the regressor, $\Phi^{\;S3}$. The calibrated output can be expressed as

(4) $$P_{calibrated}^{S3}=\Phi^{\;S3}\;\left\{P_{raw},T,\;RH,{GAS}_{raw},Hour\right\}.$$

#### 3.1.4. Scenario 4 (S4)

Both $N_{day}$ and Hour are now included as co-variates along with the raw target pollutant readings (either CO or NO_2_), the temperature and relative humidity readings and other pollutant readings from the LCS to estimate the regressor $\Phi^{\;S4}$. Therefore, the calibrated output can be written as

(5) $$P_{calibrated}^{S4}=\Phi^{\;S4}\;\left\{P_{raw},T,\;RH,{GAS}_{raw},N_{day},Hour\right\}.$$

### 3.2. Algorithm Training and Validation

As mentioned previously, we have used two machine learning algorithms, RFR and LSTM, to investigate the effects of the temporal co-variates, $N_{day}$ and Hour. A rigorous training, validation and testing method has been followed during this work. The hyperparameters have been tuned on the relevant training datasets and tested on the corresponding testing sets for the regressors. The list of the tuned hyperparameters is given in Table 2.

**Figure 2:**
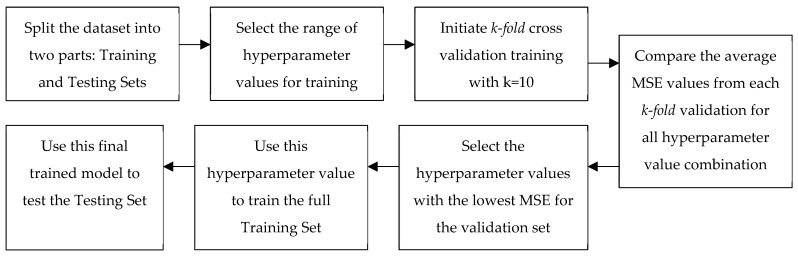
Process diagram of the dataset training, validation and testing. A *k*-fold (*k* = 10) cross-validation has been utilized to ensure that the parameters are more generalized.

A portion of each dataset (training data) is used to determine the parameter of the calibration model by training and validating the regressor model. The performance of the trained model is then evaluated on the remainder of the data (testing data) not used for training. There are two common usage situations for an LCS. In one situation, a co-located low-cost sensor can be used as a backup in case the reference grade monitor is out of commission for a short period. To emulate this situation, we split each data set so that 90% of the data were used for training/validation and 10% of the data were used for evaluating the accuracy of the trained models. We term this as Train-Test Split 1 or TTS1. The second usage situation is using the LCS after calibrating the sensors through a relatively short co-location with a reference sensor. This is emulated by using 20% of the data for training/validation and the remaining 80% for evaluating the accuracy of the trained models. We term this as Train-Test Split 2 or TTS2. The train/validation/test process has been illustrated as a diagram in Figure 2.

For the LSTM models, an early stopping method has been used during the train/validation stage. The validation sets’ MSEs are observed for each epoch. The training terminates when the MSE does not decrease by a certain tolerance threshold for a set number of epochs (patience). The weights which provide the minimum MSE within that patience are chosen as the model’s final weight.

### 3.3. Performance Metrics

Several standard performance metrics have been used in this study to evaluate the calibration models. These metrics in various ways measure the residuals or errors, i.e., difference between the calibrated output of the LCS ($P_{calibrated}$) and the ground truth reading ($P_{reference}$) for the “un-seen” test data.

Root Mean Square Error (*RMSE*), which is commonly used as a performance metric for sensor calibration [7,41,42,43,44], was utilized as a metric. *RMSE* is the standard deviation of the residuals and can be expressed as:

(6) $$RMSE=\sqrt{\frac{1}{N}\sum_{i=0}^{N-1}{\left[P_{calibrated}-P_{reference}\right]}^{2}}.$$

Here, *N* is the number of samples in the relevant test dataset.

For a more detailed investigation, we have also plotted the Cumulative Distribution Function (CDF) of absolute errors, $abs\left[P_{calibrated}-P_{reference}\right]$.

Target diagrams [26,45] were constructed for visualizing the performance of the calibration models. The *y* axis in a target diagram represents the Mean Bias Error (*MBE*) normalized by the standard deviation of the ground truth so that:

(7) $$MBE=mean\left(P_{calibrated}\right)-mean\left(P_{reference}\right),$$

(8) $$Normalised\;MBE=\frac{MBE}{\sigma_{reference}}\;.$$

Here, $\sigma_{reference}$ is the standard deviation of the ground truth for the relevant test dataset. The *x* axis of the Target Diagram represents the normalized unbiased estimate of the *RMSE*, the Centered *RMSE* (*CRMSE*), where:

(9) $$RMSE=\sqrt{{RMSE}^{2}-{MBE}^{2}},$$

(10) $$Normalised\;CRMSE=\frac{CRMSE}{\sigma_{reference}}\;.$$

Please note that the normalized *CRMSE* is multiplied by $sign\left\{\sigma_{calibrated}-\sigma_{reference}\right\}$ to produce the target diagrams, with $\sigma_{calibrated}$ being the standard deviation of the calibrated data for the relevant test dataset.

## 4. Results and Discussion

### 4.1. Model Evaluation for Different Scenarios

**Figure 3:**
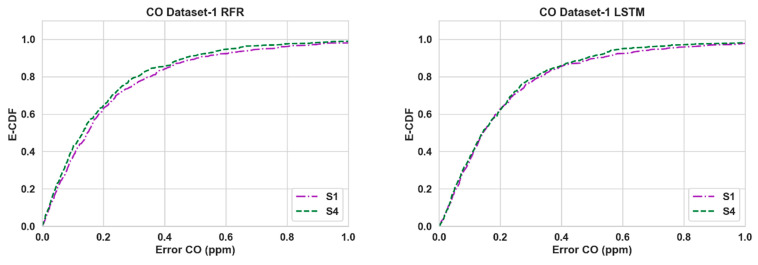

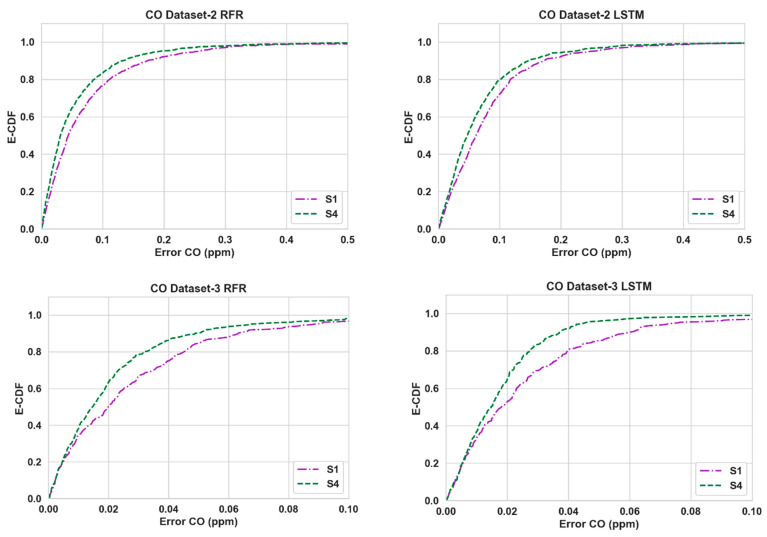
Empirical CDF plots of calibration error for CO.

**Figure 4:**
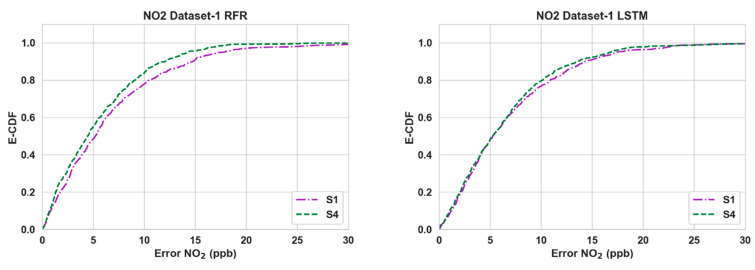

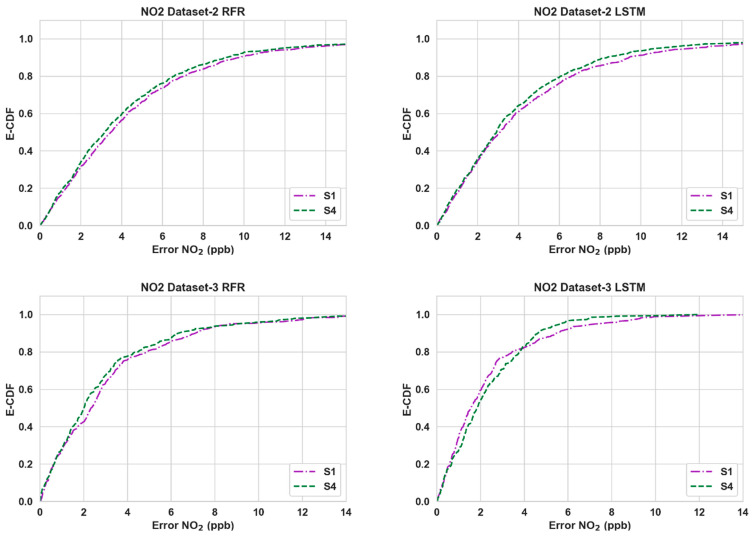
Empirical CDF plots of calibration error for NO_2_.

**Figure 5:**
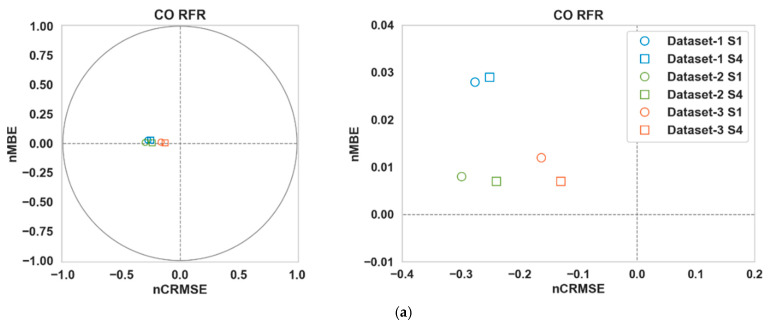

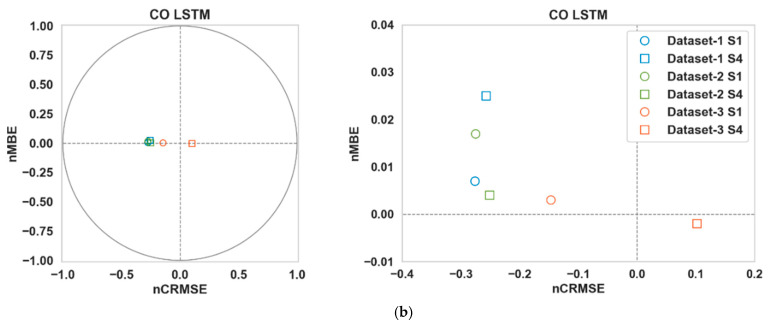
Target diagrams of (**a**) RFR and (**b**) LSTM for CO.

**Figure 6:**
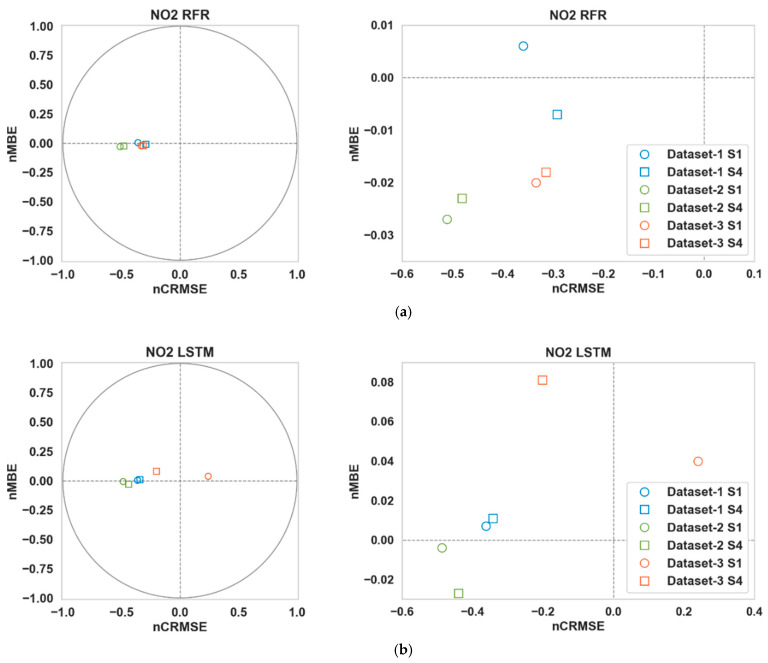
Target diagrams of (**a**) RFR and (**b**) LSTM for NO_2_.

Table 3 shows the performance of the calibration algorithms (RFR and LSTM) in different scenarios. We can make the following observations:

1. Overall, the use of $N_{day}$ and Hour has improved the calibration accuracy noticeably for both pollutants throughout all three datasets. The lowest RMSE (Table 3) is achieved for S4 in all cases.
2. For CO, the gain is quite noticeable in S2 and S4 compared to S3 for both algorithms in Datasets 2 and 3. Dataset 3 in particular showed a large improvement (around 20% or more) when $N_{day}$ was introduced as an input. For both algorithms with CO as the target pollutant, RMSE improved slightly in S3 from that of S2 in Dataset 1, while they were significantly lower (around 3% or less) in Datasets 2 and 3.
3. Overall, the improvements for NO_2_ are more modest compared to the RMSE improvements in CO. For NO_2_, these improvements were mostly below 10% in all scenarios, with the exception being RFR in S2 and S4 (more than 15%) for Dataset 1.
4. In all cases, both S2 and S4 have outperformed S3 noticeably (the only exception being CO in Dataset 1). Thus, the impact of $N_{day}$ as an input co-variate seems to be more prominent than adding Hour. However, the opposite can be seen for CO in Dataset 1.
5. The empirical CDF plots of calibration error in Figure 3 and Figure 4 show a clear improvement in S4 from S1, further demonstrating the importance of using both $N_{day}$ and Hour data as input features.
6. The target diagrams for the calibration are presented in Figure 5 and Figure 6. All the points lie inside the unit circle, i.e., radius = 1, and therefore the variance of the residuals is smaller than that of the reference measurements. Thus, the variability of the calibrated output (dependent variable) is explained by the reference data (independent variable) and not the residues. The distance of these points from the origin represents the normalized RMSE (RMSE/$\sigma_{reference}$), which shows that calibrations achieved are more accurate than the same for S1. This once again underlines the importance of adding temporal data as input features. It is also observed that the standard deviation of the calibrated data is mostly smaller than the standard deviation of the ground truth, as the majority of the points lie on the left plane.

**Table 3:** Performance analysis of RFR and LSTM in different scenarios. RMSE is in ppm for CO and ppb for NO_2_. Improvement is the decrease in RMSE for a scenario compared to the RMSE of S1 expressed in percentage.

Pollutant	Algorithm	Dataset	Parameter	Scenario			
				S1	S2	S3	S4
CO	RFR	1	RMSE	0.346	0.332	0.326	0.314
			Improvement	0	4.094	5.606	9.182
		2	RMSE	0.129	0.110	0.125	0.104
			Improvement	0	15.037	3.753	19.772
		3	RMSE	0.043	0.034	0.042	0.034
			Improvement	0	19.581	1.137	20.337
	LSTM	1	RMSE	0.344	0.335	0.326	0.322
			Improvement	0	2.63	5.17	6.44
		2	RMSE	0.119	0.110	0.117	0.109
			Improvement	0	7.54	1.58	8.66
		3	RMSE	0.039	0.029	0.038	0.027
			Improvement	0	24.91	2.97	30.89
NO_2_	RFR	1	RMSE	8.886	7.497	8.456	7.236
			Improvement	0	15.64	4.84	18.58
		2	RMSE	6.193	5.930	6.088	5.836
			Improvement	0	4.25	1.70	5.77
		3	RMSE	4.549	4.305	4.474	4.277
			Improvement	0	5.36	1.65	5.98
	LSTM	1	RMSE	8.968	8.560	8.836	8.476
			Improvement	0	4.55	1.47	5.49
		2	RMSE	5.896	5.603	5.736	5.342
			Improvement	0	4.97	2.71	9.39
		3	RMSE	8.886	7.497	8.456	7.236
			Improvement	0	15.64	4.84	18.58

In summary, using temporal parameters as co-variates for the regressors improved the calibration accuracy for both pollutants for all three datasets. The performance gain for NO_2_ is more modest compared to those achieved for CO. In general, the impact of the duration of time a sensor has been deployed is more pronounced than the time of day the reading was taken. Using both temporal co-variates (along with cross-pollutant data and temperature and relative humidity) provides the most accurate calibration for both target pollutants for all three datasets.

### 4.2. Impact of Train-Test Split

Table 4 shows the improvement in RMSE while using the temporal co-variates for a 20/80 train/test split (TTS2). This represents the use case where the LCS is co-located with a reference sensor for a set period for calibration and then afterwards deployed in the field for monitoring pollutants at locations where no AQM station is available. We can again observe noticeable improvements in S4 for both pollutants. However, the level of improvement is more modest than its 90:10 counterparts.

### 4.3. Significance of Temporal Information

Traditionally, LCS are calibrated by utilizing cross-pollutant data as co-variates alongside temperature and relative humidity data received from the LCS. However, cross-pollutant data are only available if the LCS is constructed as an array consisting of a suite of multiple pollutant sensors. Based on the efficacy of the temporal co-variates shown in this study, we believe that utilizing the number of days deployed ($N_{day}$) and time of day (Hour) data as input for the calibration algorithms may let us achieve a reasonably accurate calibration model even when the cross-pollutant data are not available.

Let us consider a scenario (termed S0) where the LCS provides only the target gas sensor data along with T and RH. We now include the two readily available co-variates (scenario S0T). We will use a similar methodology to that outlined in Section 3.2 to train and validate the algorithms for these two scenarios. RMSE improvement results for the 90:10 training and testing ratio for RFR and LSTM have been illustrated in Table 5. All the results show a noticeable improvement in RMSE. It is obvious that the accuracy of the calibration can be significantly improved even without deploying a sensor array of multiple pollutants, and therefore without increasing the cost.

**Figure 7:**
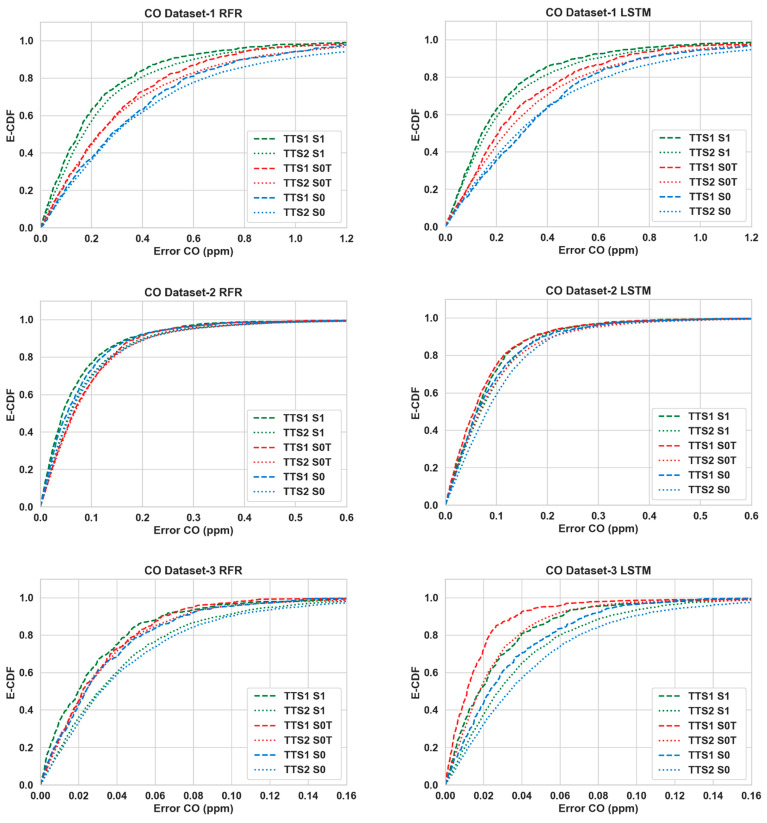
E-CDFs of CO for all three datasets for different algorithms between scenarios S0 (raw LCS + Temp + Hum), S0T (raw LCS + Temp + Hum+ *N_days_* + *Hour*), S1 (same as S1—raw LCS + Temp + Hum + other gases) and train test splits (TTS1—90:10, TTS2—20:80).

**Figure 8:**
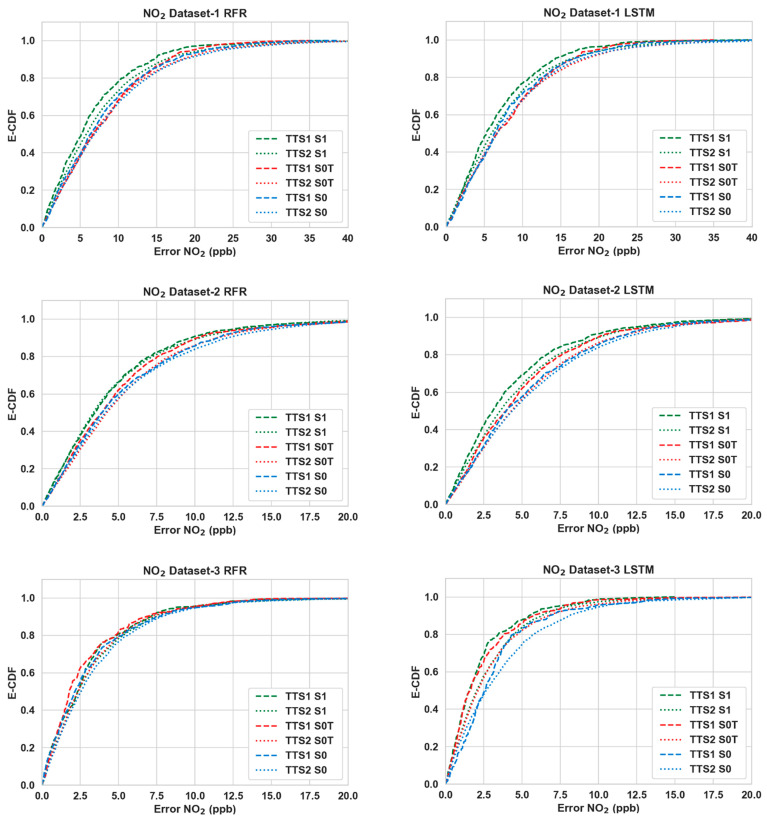
E-CDFs of NO_2_ for all three datasets for different algorithms between scenarios S0 (raw LCS + Temp + Hum), S0T (raw LCS + Temp + Hum+ *N_days_* + *Hour*) and S1 (same as S1—raw LCS + Temp + Hum + other gases) and train test splits (TTS1—90:10, TTS2—20:80).

The improvement of RMSE scores in S1 and S0T from S0 for the 90:10 training and testing ratio are shown in Table 6 and Table 7. This helps us compare the impact of temporal co-variates against that of cross-pollutant data. Table 8 and Table 9 show the comparative results for 20:80 training and testing ratio. Overall, the improvements that can be achieved with the temporal co-variates exclusively are substantial and not far behind the improvements observed when cross-pollutant data were available (and temporal co-variates were not used). The empirical CDF plots for S1 and S0T presented in Figure 7 and Figure 8 show similar encouraging patterns.

## 5. Conclusions and Future Work

In this article, we proposed to utilize temporal co-variates, namely the duration of time a sensor has been deployed and the time of day the reading was taken, to improve the calibration of low-cost sensors. For our study, we selected two common machine learning-based algorithms, Random Forest, and LSTM, and three datasets of ambient gas pollutant collected by researchers. The target pollutants of the study were CO and NO_2_. Based on our investigation, it can be concluded that the temporal co-variates can improve the calibration accuracy significantly. This is a significant outcome, as this can be achieved with readily available information.

Continual progress in deep learning presents the opportunity to use new and advanced ML algorithms. Our preliminary investigation shows that the temporal co-variates improve the accuracy of a wide range of ML methods, e.g., Gradient Boost, One Dimensional Convolutional Neural Network, Multilayer Perceptron or Artificial Neural Network, etc. However, further investigation is necessary; therefore, future research can investigate the impact of the temporal co-variates on other machine learning-based calibration algorithms. Our work shows the efficacy of various co-variates. The extent of the impact varies, potentially due to both the hardware used and the ambient conditions. We believe that the gradual degradation of the sensor’s performance, in large part, depends on the hardware. Therefore, the co-variates used in this study should improve the performance of LCSs in general. However, the degree of the efficacy would be dependent on the hardware, among other factors. A future study can investigate this issue with data collected from a diverse group of LCS hardware.

Our investigation showed that the time of deployment and time of the day have a significant impact when used as input. However, there are other available temporal parameters, such as month of the year, whether the day is a weekday or weekend, etc. While these parameters were found to have no noticeable impact for the three datasets in this work, a future study with other datasets may show them to be useful co-variates. It is also not clear how the ML models behave if the trained model from one LCS is used to calibrate another LCS with similar hardware and a similar configuration. It will be worthwhile to investigate how a transfer calibration approach can be used in such a scenario.

## Figures and Tables

**Table 1:** Details of the utilized datasets. The multisensory LCSs and the reference sensors have missing readings at some time instants for each dataset. If any reading from the LCSs or the reference sensors were missing, all data for that time instant have been removed before training the models. All three datasets have a sampling rate of 1 h. However, Dataset 3 is also available at a per-minute sampling rate. For Datasets 2 and 3, both working and auxiliary electrode data were available from the LCS.

Dataset	Time Span	Location	Number of Samples	LCS Array	Pollutant Measured	Reference Sensor
1 [23]	10/03/2004–04/04/2005	Lombardy Region, Italy	6941 (CO) 6743 (NO_2_)	MOX	CO, NO_2_, O_3_, NMHC, NO_X_	Air pollution analyzer, operated by the Regional Environmental Protection Agency (ARPA)
2 [10]	04/05/2018–24/11/2020	Naples, Italy	13,595 (CO) 12,123 (NO_2_)	EC	CO, NO_2_, O_3_	Teledyne 300 CO analyzer and Teledyne T200 NO_2_ chemiluminescence analyzer
3 [7]	01/10/2018–01/03/2019	Guangzhou, China	3639 (CO) 3412 (NO_2_)	EC	CO, NO_2,_ O_3_	CO data were collected by a gas analyzer based on infrared absorption (Model 48i-TLE, Thermo Scientific, Waltham, MA, USA). NO_2_ was measured by a chemiluminescence analyzer (Model 42i-TL, Thermo Scientific, Waltham, MA, USA)

**Table 2:** List of hyperparameters that were tuned for RFR and LSTM.

Algorithm	List of Hyperparameters
RFR	Maximum depth of tree, maximum number of leaf nodes, number of trees in the forest.
LSTM	Number of LSTM layers, time steps, number of units in the LSTM layers, activation function, dropout rate in dropout layers, learning rate of the optimizer, batch size.

**Table 4:** Performance improvement of RMSE in S4 from S1 for 20:80 split (TTS2).

Pollutant	Algorithm	Improvement of RMSE in S4 from S1 (in %)		
		Dataset 1	Dataset 2	Dataset 3
CO	RFR	4.49	11.95	8.92
	LSTM	6.34	4.13	19.24
NO_2_	RFR	13.19	3.93	2.73
	LSTM	4.49	3.55	2.36

**Table 5:** Performance improvement of RMSE resulting from temporal co-variates only for the 90:10 split.

Pollutant	Algorithm	Improvement of RMSE in S0T from S0 (in %)		
		Dataset 1	Dataset 2	Dataset 3
CO	RFR	15.75	0.76	10.86
	LSTM	15.57	2.31	11.11
NO_2_	RFR	3.12	5.36	10.09
	LSTM	1.85	5.21	20.91

**Table 6:** Improvement of RMSE in S1 (raw + temperature + humidity + other gases) and S0T (raw + temperature + humidity + $N_{day}$ + Hour) from S0 (raw + temperature + humidity) for CO in a 90:10 split.

Algorithm	Scenario	Improvement of CO RMSE in S1 and S0T from S0 (in %)		
		Dataset 1	Dataset 2	Dataset 3
RFR	S1	31.89	1.83	6.78
	S0T	15.75	0.76	10.86
LSTM	S1	31.41	8.58	14.48
	S0T	15.57	2.31	11.11

**Table 7:** Improvement of RMSE in S1 (raw + temperature + humidity + other gases) and S0T (raw + temperature + humidity + $N_{day}$ + Hour) from S0 (raw + temperature + humidity) for NO_2_ in a 90:10 split.

Algorithm	Scenario	Improvement of NO_2_ RMSE in S1 and S0T from S0 (in %)		
		Dataset 1	Dataset 2	Dataset 3
RFR	S1	15.38	12.63	6.18
	S0T	3.12	5.36	10.09
LSTM	S1	13.49	16.73	29.59
	S0T	1.85	5.21	20.91

**Table 8:** Improvement of RMSE in S1 (raw + temperature + humidity + other gases) and S0T (raw + temperature + humidity + $N_{day}$ + Hour) from S0 (raw + temperature + humidity) for CO in 20:80 split.

Algorithm	Scenario	Improvement of CO RMSE in S1 and S0T from S0 (in %)		
		Dataset 1	Dataset 2	Dataset 3
RFR	S1	31.99	2.72	6.25
	S0T	17.34	0.38	21.88
LSTM	S1	29.19	8.84	14.52
	S0T	16.26	−0.78	20.97

**Table 9:** Improvement of RMSE in S1 (raw + temperature + humidity + other gases) and S0T (raw + temperature + humidity + $N_{day}$ + Hour) from S0 (raw + temperature + humidity) for NO_2_ in 20:80 split.

Algorithm	Scenario	Improvement of NO_2_ RMSE in S1 and S0T from S0 (in %)		
		Dataset 1	Dataset 2	Dataset 3
RFR	S1	12.13	15.04	4.92
	S0T	3.30	4.52	7.53
LSTM	S1	7.52	13.93	18.51
	S0T	−0.46	4.72	15.28

## Data Availability

Dataset 1 is open access and can be found here: https://archive.ics.uci.edu/ml/datasets/Air+Quality# (accessed on 12 August 2020). Dataset 2 and 3 were collected from De Vito et al. and Liang et al., respectively.

## References

[1] World Health Organization (2021). WHO Global Air Quality Guidelines: Particulate Matter (PM2.5 and PM10), Ozone, Nitrogen Dioxide, Sulfur Dioxide and Carbon Monoxide.

[2] Cohen A.J., Brauer M., Burnett R., Anderson H.R., Frostad J., Estep K., Balakrishnan K., Brunekreef B., Dandona L., Dandona R. (2017). Estimates and 25-year trends of the global burden of disease attributable to ambient air pollution: An analysis of data from the Global Burden of Diseases Study 2015. Lancet.

[3] Kampa M., Castanas E. (2008). Human health effects of air pollution. Environ. Pollut..

[4] Alshamsi A., Anwar Y., Almulla M., Aldohoori M., Hamad N., Awad M. Monitoring pollution: Applying IoT to create a smart environment. Proceedings of the 2017 International Conference on Electrical and Computing Technologies and Applications (ICECTA).

[5] Tsujita W., Yoshino A., Ishida H., Moriizumi T. (2005). Gas sensor network for air-pollution monitoring. Sens. Actuators B Chem..

[6] Ali S., Glass T., Parr B., Potgieter J., Alam F. (2020). Low Cost Sensor with IoT LoRaWAN Connectivity and Machine Learning-Based Calibration for Air Pollution Monitoring. IEEE Trans. Instrum. Meas..

[7] Liang Y., Wu C., Jiang S., Li Y.J., Wu D., Li M., Cheng P., Yang W., Cheng C., Li L. (2021). Field comparison of electrochemical gas sensor data correction algorithms for ambient air measurements. Sens. Actuators B Chem..

[8] Topalović D.B., Davidović M.D., Jovanović M., Bartonova A., Ristovski Z., Jovašević-Stojanović M. (2019). In search of an optimal in-field calibration method of low-cost gas sensors for ambient air pollutants: Comparison of linear, multilinear and artificial neural network approaches. Atmos. Environ..

[9] De Vito S., Esposito E., Massera E., Formisano F., Fattoruso G., Ferlito S., Del Giudice A., D’Elia G., Salvato M., Polichetti T. (2021). Crowdsensing IoT Architecture for Pervasive Air Quality and Exposome Monitoring: Design, Development, Calibration, and Long-Term Validation. Sensors.

[10] De Vito S., Di Francia G., Esposito E., Ferlito S., Formisano F., Massera E. (2020). Adaptive machine learning strategies for network calibration of IoT smart air quality monitoring devices. Pattern Recognit. Lett..

[11] Shaban K.B., Kadri A., Rezk E. (2016). Urban Air Pollution Monitoring System with Forecasting Models. IEEE Sens. J..

[12] Liu X., Cheng S., Liu H., Hu S., Zhang D., Ning H. (2012). A survey on gas sensing technology. Sensors.

[13] Maag B., Zhou Z., Thiele L. (2018). A Survey on Sensor Calibration in Air Pollution Monitoring Deployments. IEEE Internet Things J..

[14] Yi W., Lo K., Mak T., Leung K., Leung Y., Meng M. (2015). A Survey of Wireless Sensor Network Based Air Pollution Monitoring Systems. Sensors.

[15] Jiao W., Hagler G., Williams R., Sharpe R., Brown R., Garver D., Judge R., Caudill M., Rickard J., Davis M. (2016). Community Air Sensor Network (CAIRSENSE) project: Evaluation of low-cost sensor performance in a suburban environment in the southeastern United States. Atmos. Meas. Tech..

[16] Badura M., Batog P., Drzeniecka-Osiadacz A., Modzel P. (2022). Low- and Medium-Cost Sensors for Tropospheric Ozone Monitoring—Results of an Evaluation Study in Wroclaw, Poland. Atmosphere.

[17] Hofman J., Nikolaou M., Shantharam S.P., Stroobants C., Weijs S., La Manna V.P. (2022). Distant calibration of low-cost PM and NO_2_ sensors; evidence from multiple sensor testbeds. Atmos. Pollut. Res..

[18] Rogulski M., Badyda A., Gayer A., Reis J. (2022). Improving the Quality of Measurements Made by Alphasense NO_2_ Non-Reference Sensors Using the Mathematical Methods. Sensors.

[19] Zuidema C., Schumacher C.S., Austin E., Carvlin G., Larson T.V., Spalt E.W., Zusman M., Gassett A.J., Seto E., Kaufman J.D. (2021). Deployment, Calibration, and Cross-Validation of Low-Cost Electrochemical Sensors for Carbon Monoxide, Nitrogen Oxides, and Ozone for an Epidemiological Study. Sensors.

[20] Cordero J.M., Borge R., Narros A. (2018). Using statistical methods to carry out in field calibrations of low cost air quality sensors. Sens. Actuators B Chem..

[21] Djedidi O., Djeziri M.A., Morati N., Seguin J.-L., Bendahan M., Contaret T. (2021). Accurate detection and discrimination of pollutant gases using a temperature modulated MOX sensor combined with feature extraction and support vector classification. Sens. Actuators B Chem..

[22] Bigi A., Mueller M., Grange S.K., Ghermandi G., Hueglin C. (2018). Performance of NO, NO_2_ low cost sensors and three calibration approaches within a real world application. Atmos. Meas. Tech..

[23] De Vito S., Esposito E., Salvato M., Popoola O., Formisano F., Jones R., Di Francia G. (2018). Calibrating chemical multisensory devices for real world applications: An in-depth comparison of quantitative machine learning approaches. Sens. Actuators B Chem..

[24] Esposito E., De Vito S., Salvato M., Fattoruso G., Bright V., Jones R.L., Popoola O. (2018). Stochastic Comparison of Machine Learning Approaches to Calibration of Mobile Air Quality Monitors.

[25] Esposito E., De Vito S., Salvato M., Fattoruso G., Di Francia G. (2017). Computational Intelligence for Smart Air Quality Monitors Calibration.

[26] Zimmerman N., Presto A.A., Kumar S.P., Gu J., Hauryliuk A., Robinson E.S., Robinson A.L., Subramanian R. (2018). A machine learning calibration model using random forests to improve sensor performance for lower-cost air quality monitoring. Atmos. Meas. Tech..

[27] Bagkis E., Kassandros T., Karatzas K. (2022). Learning Calibration Functions on the Fly: Hybrid Batch Online Stacking Ensembles for the Calibration of Low-Cost Air Quality Sensor Networks in the Presence of Concept Drift. Atmosphere.

[28] Bittner A.S., Cross E.S., Hagan D.H., Malings C., Lipsky E., Grieshop A.P. (2022). Performance characterization of low-cost air quality sensors for off-grid deployment in rural Malawi. Atmos. Meas. Tech..

[29] Malings C., Tanzer R., Hauryliuk A., Kumar S.P., Zimmerman N., Kara L.B., Presto A.A., Subramanian R. (2019). Development of a general calibration model and long-term performance evaluation of low-cost sensors for air pollutant gas monitoring. Atmos. Meas. Tech..

[30] Borrego C., Ginja J., Coutinho M., Ribeiro C., Karatzas K., Sioumis T., Katsifarakis N., Konstantinidis K., De Vito S., Esposito E. (2018). Assessment of air quality microsensors versus reference methods: The EuNetAir Joint Exercise—Part II. Atmos. Environ..

[31] Fonollosa J., Sheik S., Huerta R., Marco S. (2015). Reservoir computing compensates slow response of chemosensor arrays exposed to fast varying gas concentrations in continuous monitoring. Sens. Actuators B Chem..

[32] Balabin R.M., Lomakina E.I. (2011). Support vector machine regression (SVR/LS-SVM)—An alternative to neural networks (ANN) for analytical chemistry? Comparison of nonlinear methods on near infrared (NIR) spectroscopy data. Analyst.

[33] Sheik S., Marco S., Huerta R., Fonollosa J. (2014). Continuous prediction in chemoresistive gas sensors using reservoir computing. Procedia Eng..

[34] Wang S., Hu Y., Burgués J., Marco S., Liu S.-C. Prediction of gas concentration using gated recurrent neural networks. Proceedings of the 2020 2nd IEEE International Conference on Artificial Intelligence Circuits and Systems (AICAS).

[35] Han P., Mei H., Liu D., Zeng N., Tang X., Wang Y., Pan Y. (2021). Calibrations of low-cost air pollution monitoring sensors for CO, NO_2_, O_3_, and SO_2_. Sensors.

[36] Wei P., Sun L., Anand A., Zhang Q., Huixin Z., Deng Z., Wang Y., Ning Z. (2020). Development and evaluation of a robust temperature sensitive algorithm for long term NO_2_ gas sensor network data correction. Atmos. Environ..

[37] Esposito E., De Vito S., Salvato M., Bright V., Jones R.L., Popoola O. (2016). Dynamic neural network architectures for on field stochastic calibration of indicative low cost air quality sensing systems. Sens. Actuators B Chem..

[38] Hu K., Sivaraman V., Luxan B.G., Rahman A. (2016). Design and Evaluation of a Metropolitan Air Pollution Sensing System. IEEE Sens. J..

[39] Idrees Z., Zou Z., Zheng L. (2018). Edge Computing Based IoT Architecture for Low Cost Air Pollution Monitoring Systems: A Comprehensive System Analysis, Design Considerations & Development. Sensors.

[40] Ali S., Alam F., Arif K.M., Potgieter J. (2023). Low-Cost CO Sensor Calibration Using One Dimensional Convolutional Neural Network. Sensors.

[41] Zhu S., Lian X., Liu H., Hu J., Wang Y., Che J. (2017). Daily air quality index forecasting with hybrid models: A case in China. Environ. Pollut..

[42] Zhu J., Wu P., Chen H., Zhou L., Tao Z. (2018). A Hybrid Forecasting Approach to Air Quality Time Series Based on Endpoint Condition and Combined Forecasting Model. Int. J. Environ. Res. Public Health.

[43] Wang P., Liu Y., Qin Z., Zhang G. (2015). A novel hybrid forecasting model for PM_10_ and SO_2_ daily concentrations. Sci. Total Environ..

[44] Jiang P., Li C., Li R., Yang H. (2019). An innovative hybrid air pollution early-warning system based on pollutants forecasting and Extenics evaluation. Knowl.-Based Syst..

[45] Spinelle L., Gerboles M., Villani M.G., Aleixandre M., Bonavitacola F. (2017). Field calibration of a cluster of low-cost commercially available sensors for air quality monitoring. Part B: NO, CO and CO_2_. Sens. Actuators B Chem..

